# Narrative Review on Common Traits of Parkinson’s Disease and Epilepsy

**DOI:** 10.3390/jcm14082716

**Published:** 2025-04-15

**Authors:** Christian Tilz, Ying Wang-Tilz

**Affiliations:** 1 Epilepsy Center Bodensee, Clinik of Neurology and Epileptology, ZfP Südwürttemberg, Weingartshofer Str. 2, 88214 Ravensburg, Germany; 2 Department of Neurology, University Hospital of Graz, Univesity Graz, Auenbruggerplatz 22, 8036 Graz, Austria; 3 Hospital of Barmherzigen Brüder Regensburg, Clinik of Neurology, Prüfeninger Str. 86, 93049 Regensburg, Germany; ying.wang-tilz@barmherzige-regensburg.de

**Keywords:** epilepsy, Parkinson’s disease, antiseizure medication (ASM), genetics, neurostimulation

## Abstract

Epilepsy and Parkinson’s disease (PD) are two common neurological disorders, with a lifetime prevalence of approximately 1% and 0.4%, respectively. Both conditions affect movement and brain function and were traditionally considered distinct, with different pathophysiological mechanisms. However, recent research suggests potential links between them. Some studies indicate that epilepsy may contribute to the development of PD due to chronic neuroinflammation, excitotoxicity, and neuronal loss. Conversely, PD-related neurodegeneration in dopaminergic pathways might increase susceptibility to seizures. This article presents a narrative review of the limited literature on the pathophysiological mechanisms linking epilepsy and PD, including shared genetic factors, neurodegenerative processes, and alterations in the neurotransmitter system. It also examines the influence of anti-seizure medications and dopaminergic treatments on the symptoms and progression of both disorders, as well as their common clinical features. Additionally, the limitations of the existing data on this topic are discussed. Understanding the true relationship between these two disorders is crucial, as it could provide insight into common neurobiological mechanisms and lead to improved therapeutic strategies.

## 1. Introduction

Epilepsies and epileptic disorders have a lifetime prevalence of about 1%, making them the most common chronic neurological diseases. Although there are various etiologies for the development of epileptic disorders—such as genetic, traumatic, tumorous, or vascular causes—the shared pathophysiological mechanism at the cellular level is a hypersynchronization of neuronal activity, which can be either focal or generalized.

Based on seizure origin, there are three main groups of epilepsies according to the classification of the International League Against Epilepsy (ILAE) [1]. These are generalized epilepsies, which account for about one-third of epileptic syndromes, have a highly variable phenotype, and are often associated with a genetic background; focal epilepsies, which are primarily caused by focal brain insults such as ischemic stroke, hemorrhagic bleeding, post-traumatic lesions, or other structural changes (e.g., due to dementia); and epilepsies of unknown origin, which represent a small subset of epileptic disorders.

Parkinson’s disease (PD) is a progressive neurodegenerative disorder that primarily affects movement. It is caused by the loss of dopamine-producing neurons in the substantia nigra, a brain region responsible for movement control. The exact cause of this neuronal loss is not fully understood, but it is believed to result from a combination of genetic and environmental factors.

In recent years, advances in the understanding of the pathophysiological mechanisms of Parkinson’s disease have led to a new classification, published in 2024 by Höglinger et al. [2] for research purposes. This classification, known as SynNeurGe, categorizes PD based on three main etiological factors: (1) PD associated with pathological aggregation of α-synuclein, as seen in multiple system atrophy; (2) PD with evidence of underlying neurodegeneration, identified through neuroimaging procedures; and (3) PD with documented pathogenic gene variants.

Epilepsy and PD have distinct causes and mechanisms; however, research suggests a possible relationship between them. Although analyses using large databases have yielded some findings, most published clinical studies are observational, retrospective, and descriptive, often based on small sample sizes or even individual case reports. This is also true for research on potential common pathophysiological mechanisms, most of which come from animal experiments.

This article aims to provide a narrative overview, beginning with a brief summary of epilepsy and PD, followed by an exploration of their common features. It traces published findings from 1990 to 2024, focusing on shared pathophysiology, clinical relationships, and common therapeutic approaches between epilepsy and PD.

Although the available data remain insufficient to draw definitive conclusions, this article seeks to highlight the topic, encouraging greater attention and further studies to clarify the true relationship between these two common neurological disorders for improved clinical prevention and treatment.

## 2. Epilepsy

### 2.1. Definition and Classification

Epilepsy is defined by the International League Against Epilepsy (ILAE) as a brain disorder characterized by a persistent predisposition to generate epileptic seizures, along with neurobiological, cognitive, psychological, and social consequences (Fisher et al., 2014) [1]. According to the ILAE classification, seizures are categorized based on their etiology into structural, genetic, infectious, metabolic, immune, and unknown origin.

### 2.2. Pathophysiology

Epileptic seizures arise from an imbalance between excitatory (glutamatergic) and inhibitory (GABAergic) neurotransmission at the cellular level. On one hand, genetic factors can lead to global neuronal hyperexcitability, which typically affects the entire cortex simultaneously and most often results in primary generalized epilepsies [3]. On the other hand, structural brain damage—such as traumatic brain injuries, vascular lesions, or inflammatory causes—can lead to focal epilepsies, which originate from damage to a specific cortical area.

The temporal lobe is the most commonly affected region in focal epilepsies, followed by the frontal lobe. Some structural lesions may be congenital (e.g., hippocampal sclerosis, cortical dysplasia), but the majority are acquired over a person’s lifetime, particularly in the elderly. With increasing life expectancy, structural epilepsies in older adults are becoming a growing contributor to the global burden of epilepsy [4].

Genetic epilepsies can result from channelopathies, involving dysfunctions in ion channels such as sodium, potassium, and calcium channels. Key mechanisms may include abnormalities in voltage-gated sodium, potassium, and calcium channels. To date, more than 500 genes have been identified in relation to epilepsy [5], including mutations in SCNA1, SCL2A1, KCNQ1, CDKL5, and DEPDC5.

#### 2.2.1. Voltage Gated Sodium Channels (VGSC)

One of the best-known examples of channelopathies involved in several neurological disorders is voltage-gated sodium channelopathies, with particular regard to **SCN** mutations. **SCN1A** mutations have been described in more than 80% of cases associated with Dravet syndrome. Depending on the type of mutation (gain-of-function or loss-of-function), the phenotype of **SCN1A** mutations can cause not only different kinds of epilepsy, such as genetic epilepsies with febrile seizures plus (GEFS+), but also infantile developmental and epileptic encephalopathy (DEE) with movement disorders [6].

**SCN** mutations have also been found in the context of several other neurological conditions, such as Parkinson’s disease, multiple sclerosis, Alzheimer’s disease, and autism spectrum disorders [7]. It has been reported that Parkinson’s disease may be associated with **SCN1A** mutations as well as with **SCN3A** mutations, which can also lead to severe developmental and epileptic encephalopathies [8].

#### 2.2.2. SCL2A1 Gene

Another representative example of genetic epilepsies is Glucose Transporter Type 1 Deficiency Syndrome (GLUT1-DS), which is caused by a mutation in **SCL2A1**. It is known that mutations in **SCL2A1** can lead to a broad spectrum of other epilepsy syndromes, such as myoclonic astatic epilepsy (Doose syndrome), early-onset absence epilepsy (EOAE), and childhood absence epilepsy. Furthermore, it has been reported that mutations in this gene can also cause several types of movement disorders, including paroxysmal exercise-induced dyskinesias (PED) [9].

Although the genetic factors underlying epilepsy and Parkinson’s disease are very different, there are some genes that are associated with both diseases. In particular, factors leading to inflammation may help explain common etiological pathways [10].

#### 2.2.3. Tuberose Sclerosis Proteins 1 and 2 (TSC1/TSC2)

The deletion, rearrangement, or inactivating mutations of the tumor-suppressor genes **TSC1** or **TSC2** can lead to uncontrolled expression of the proteins hamartin and tuberin. This results in manifestations in several organs, particularly cutaneous manifestations with typical skin lesions, renal involvement with angiomyolipomas (benign tumors that occur in up to 80% of TSC patients), and various cerebral manifestations, including cortical and subcortical tubers and cortical dysplasia. These brain abnormalities lead to different types of epileptic seizures, most commonly infantile spasms and focal seizures, which often begin very early in childhood [11].

Since 2017, the introduction of everolimus has provided a precision-based curative treatment option, not only aimed at seizure reduction but also serving as a disease-modifying therapy. This treatment works by reconstituting the regulation of the TSC protein complex through the inhibition of the overexpressed mTOR pathway [12]. This is an example of drug selection based on genetically identified epilepsy syndromes, offering individualized treatment possibilities.

### 2.3. Epidemiology

Epilepsy affects approximately 50–70 million people globally, with a higher prevalence in low- and middle-income countries [13].

### 2.4. Diagnosis

For a correct diagnosis of epilepsy, including the type of seizure, a detailed description of seizure semiology from both the patient and any witnesses is indispensable. Such information can provide valuable insight for accurate localization and focalization of the seizure origin. For example, the sensation of an aura can be a reliable and strong indicator for identifying the seizure type and the probable focus of seizure origin. Other important factors include the lateralized semiological onset of the seizure (which typically occurs contralaterally to the origin of the focus), the duration of the seizure (epileptic seizures generally last around 30 s for focal non-awareness seizures and 2–3 min for focal to generalized tonic–clonic seizures), and the circadian pattern (frontal lobe epilepsies primarily occur during the second half of the night, while primary generalized epilepsies often occur upon awakening in the morning).

Cardiac phenomena, such as ictal tachycardia, are also common in epilepsy (with 80% of temporal lobe epilepsies associated with ictal tachycardia), and they can be detected by the new generation of vagus nerve stimulators, which are used for automatic seizure detection [14].

The EEG is the most important diagnostic tool for proper diagnosis and understanding the pathophysiology of epilepsies. More than 100 years after the first recording of bioelectrical currents by Hans Berger, it remains the only tool that can reveal hypersynchronization of neuronal activity and is, therefore, indispensable for the accurate diagnosis of epilepsies and epileptic syndromes to this day [15]. It is also the only method that allows differentiation between focal and generalized epilepsy syndromes.

### 2.5. Treatment

#### 2.5.1. Anti-Seizure Medication (ASM)

In general, the first-line treatment for epilepsy is pharmacological, using anti-seizure drugs (ASDs) that work to suppress the hyperexcitability of epileptic neurons. These drugs do not remove the underlying cause of epilepsy but help to suppress the hypersynchronization of neurons, thus improving the imbalance in neuronal hyperexcitability. For this reason, they are referred to as anti-seizure medications (ASMs). They can be used either in monotherapy or in combination.

The development of ASDs has evolved over more than a century, starting with the discovery of bromides in the 19th century. Phenobarbital, introduced in 1912, was the first widely used anti-seizure medication, followed by phenytoin in 1938, which offered efficacy with fewer sedative effects. Over the mid-to-late 20th century, drugs like carbamazepine, valproate, and benzodiazepines expanded treatment options.

In the late 20th and early 21st centuries, many newer ASDs were developed, including the now frequently prescribed lamotrigine, levetiracetam, and lacosamide. These drugs offered improved safety profiles, fewer side effects, and more targeted mechanisms of action. Modern research continues to explore personalized medicine, gene therapies, and non-pharmacological treatments for epilepsy.

As of the end of 2024, 30 anti-seizure medications (ASMs) are available for the treatment of epilepsy [16]. These medications vary in their mechanisms of action, efficacy, and side effect profiles, providing healthcare providers with a range of options to tailor treatment plans to individual patient needs. Even orphan drugs for rare diseases have been commercialized for individualized pharmacological approaches, such as Fenfluramine and Cannabidiol for Dravet syndrome and Lennox–Gastaut syndrome, or ganaxolone for CDKL5-mediated epilepsies [16,17,18]. Everolimus, approved in 2017 for the treatment of TSC1/TSC2-mediated epilepsies, is the first pharmacological option that not only provides symptomatic treatment but also offers the possibility of a curative therapeutic approach for tuberous sclerosis [12].

#### 2.5.2. Epilepsy Surgery and Neurostimulation

About one third of patients with epilepsy do not achieve sufficient seizure control despite treatment with two or more ASMs. Since the chance of achieving seizure freedom after two ASM treatments is relatively poor, these patients should be considered pharmacoresistant early on. For them, further treatment options are needed, such as epilepsy surgery, ranging from traditional resections to minimally invasive neurosurgical treatments like MR-guided laser interstitial thermal therapy (MRgLITT) or stereo-EEG-guided radiofrequency thermocoagulation (RF-TC) [19].

In addition, neurostimulation methods are also commonly used for patients with difficult-to-treat epilepsies who are not candidates for surgical resection. Several neurostimulation approaches are available for the treatment of pharmacoresistant epilepsies.

Among these, the first vagus nerve stimulation (VNS) was approved in Europe in 1994 before receiving FDA approval and has been in clinical application since 1995 for patients with difficult-to-treat epilepsy. VNS is the most well-known and longest-used neurostimulation method in epilepsy treatment, with more than 150,000 patients worldwide having been treated. Approximately 40–60% of patients show a significant reduction in seizures (responders). The response is typically seen three months after implantation, with further improvements possible over the next three years. With the advancement of automatic seizure detection and response capabilities, VNS can now recognize ictal tachycardia at the very onset of a seizure and provide additional stimulation to either reduce seizure severity or achieve seizure termination.

Deep brain stimulation (DBS) has also been approved in Europe since 2014 [20]. For pharmacoresistant epilepsy, the classical stimulation point is the anterior nucleus of the thalamus (N. anterior thalami). However, due to severe side effects—especially psychiatric issues such as depression, suicidal ideation, and psychosis—the use of DBS is controversial in epileptology and has not been as widely established as in the treatment of Parkinson’s disease, movement disorders, or tremor.

A very new and promising treatment option is the epicranial stimulation electrode (EASEE^®^), which involves the epicranial application of stimulation electrodes for difficult-to-treat focal epilepsies with an origin relatively close to the scalp [21].

Another novel neurostimulation option is Responsive Neurostimulation (RNS) [22]. In this approach, stimulation is delivered by one or two intracerebral EEG electrodes placed in the region of the seizure origin, which must first be localized through intracranial stereo-EEG recordings. The stimulator is placed under the scalp and is the first and only neurostimulation method with a closed-loop stimulation mode. It continuously records EEG activity and delivers stimulation immediately when ictal EEG activity is detected. The seizure reduction rate with RNS is 68%. RNS is currently only available in the US, where it is approved not only for pharmacoresistant epilepsies but also for chronic pain.

Transcutaneous VNS (tVNS) is an interesting non-invasive approach involving stimulation of the auricular branch of the vagus nerve. After a proof-of-concept trial [23], where a daily application of 3 h was tested, several research groups have continued working on this technique. However, it has not been commercialized yet [23,24,25,26,27].

#### 2.5.3. Antiseizure Medications (ASM) and the Risk of Parkinson’s Disease

The question of whether exposure to antiseizure medications (ASMs) is correlated with a higher incidence of Parkinson’s disease has been raised. However, publications on this issue are scarce, with most of them based on clinical observations without control groups and others relying on animal models [28,29,30,31,32].

A case–control study using data from the UK Biobank (UKB) investigated the association between the use of ASMs and the prevalence of Parkinson’s disease in 1433 patients using four different ASMs (carbamazepine, lamotrigine, levetiracetam, and sodium valproate) compared to 8598 controls. Data collection occurred between 2006 and 2010 from participants in the UKB longitudinal cohort study, aged between 40 and 69 years, with data extraction on 30 June 2021. In this study, cases were defined as individuals with a Hospital Episode Statistics (HES)-coded diagnosis of Parkinson’s disease. Controls were matched 6:1 for potential risk factors for Parkinson’s disease such as age, sex, race, ethnicity, and socioeconomic status. Cases of transient drug-induced parkinsonism were excluded by withdrawing patients who had prescriptions issued within 1, 2, and 5-year windows prior to the index date. ASM prescription records were considered only before the diagnosis of Parkinson’s disease.

The study showed that prescription of an ASM was associated with an increased risk of subsequent incident Parkinson’s disease, with an odds ratio of 1.80 (95% CI: 1.35–2.40). Among the ASMs, lamotrigine, levetiracetam, and valproate showed stronger associations with Parkinson’s disease, with valproate having the highest rate and carbamazepine the lowest [33]. The authors concluded that a higher prevalence of Parkinson’s disease was correlated with ASM exposure. They suggested that this could be due to the interference of ASMs with dopamine metabolism. However, several limitations of this observational study were discussed, such as diagnostic bias from using HES data and selection bias. One major limitation was the lack of information on the etiology of epilepsy in the patients and the etiology of those with both epilepsy and Parkinson’s disease. In addition to brain disorders, factors such as increased age and comorbidities may contribute to the coexistence of Parkinson’s disease, rather than ASMs alone. This is a significant limitation in interpreting the relationship between ASMs and Parkinson’s disease.

Therefore, direct evidence on the relationship between ASMs and the development of Parkinson’s disease is still lacking. Further studies, with stratification of all possible cofactors and biases and larger sample sizes—including data on the newer generation of ASMs—are urgently needed.

## 3. Parkinson’s Disease (PD)

Parkinson’s disease (PD) is a neurological disorder that primarily affects the elderly, with a prevalence of 0.4% across the lifespan and 1% of the population over 60 years of age [31]. It is caused by the degeneration of dopaminergic neurons in the substantia nigra of the striatum (Hammonds). Described by James Parkinson in 1817 as “shaking palsy”, PD was traditionally considered a purely neurodegenerative disorder. Although the exact etiology of PD remains unclear, both genetic and environmental factors are thought to play essential roles [32]. Elevated levels of synchronous oscillations in the basal ganglia neurons are crucial to the pathophysiology of PD [33,34]. Voltage-gated sodium channels (VGSC) may also play a significant role in the electrical imbalance of neurons in PD [35].

Advances in understanding the etiologic factors have led to a revised classification in 2024, which divides PD according to neurodegeneration, genetic findings, and the presence of synuclein.

### 3.1. Genetics of PD

As mentioned above, genetics play a fundamental role in the classification and diagnosis of Parkinson’s disease. Genetic testing should be recommended for younger patients with Parkinson’s disease (onset under the age of 60), especially if one or more family members are also affected by PD. This testing provides valuable information regarding prognosis, the risk for family members, and therapeutic decisions. Considerable attention is currently being given to therapeutic approaches in PD in relation to genetic variants. Tremendous efforts are being made to develop new approaches for disease-modifying treatments, rather than just symptomatic treatments.

As with epilepsy, there are various modes of genetic inheritance in PD. Monogenic pathogenic variants predisposing individuals to Parkinson’s disease can be identified in approximately 10–15% of patients with PD. Patients with gene mutations such as GBA1, LRRK2, or SNCA exhibit typical histopathological signs of Lewy bodies, which are characteristic of PD [2].

The GBA1 mutation is a well-known risk factor for PD. Carriers of this mutation have up to a fivefold higher risk of developing PD by the age of 60. The GBA1 gene encodes the enzyme glucocerebrosidase and is also associated with Gaucher’s disease. Small studies examining the use of ambroxol, an amylolytic drug, have shown improvement in Parkinson’s patients with this mutation [36].

Other genetic mutations associated with a similar clinical presentation of PD include mutations in VPS35, DJ1, and PARKIN, which should be classified separately from variants with typical histopathological findings. According to the consensus group of the new PD classification [2], it is suggested to classify these as Parkinson’s syndrome with the corresponding genetic mutation (e.g., PARK-VPS35).

The LRRK2 gene mutation has incomplete penetrance, affecting 1–2% of people with PD. There are also personalized therapeutic approaches for LRRK2 mutations. Trials with LRRK2 kinase inhibitors have shown therapeutic efficacy in small clinical trials, providing a potential personalized therapeutic approach [37].

### 3.2. Diagnosis

- Clinical Examination: The assessment of movement patterns, UPDRS scoring, and the Dopa test are essential for the classification and staging of Parkinson’s disease (PD).
- DAT Scan: A DAT scan should be performed early after the first diagnosis of PD to confirm nigrostriatal degeneration for the differential diagnosis of unclear tremors or Parkinsonism, especially if a therapeutic decision will be based on this information.
- FDG-PET: An FDG-PET can be performed to estimate the risk of developing dementia.
- **Genetic Testing**: As mentioned above, genetic testing plays an important role in diagnosing PD, particularly for younger patients and those with a family history of the disease.

### 3.3. Treatment

#### 3.3.1. Pharmacological Treatment

In the treatment of Parkinson’s disease (PD), medications such as levodopa, DOPA agonists (non-ergotamine), MAO inhibitors, and COMT inhibitors are initially used in monotherapy, and then in combination as the disease progresses. Over time, patients typically experience significant fluctuations in the efficacy of these medications, despite various formulations of oral levodopa or dopamine agonists. Additionally, the risk of hyperkinetic or hypokinetic periods increases substantially.

Invasive Treatment Options: Continuous infusion via gastrojejunostomy (GJ) application is a therapeutic approach that allows for more consistent substitution throughout the day. With the introduction of phosphorylated Foslevodopa/Foscarbidopa (Produodopa^®^), a subcutaneous infusion is now possible via a pump system. Compared to previous pump systems that required gastrointestinal application, this novel subcutaneous infusion method permits easier and more practical continuous levodopa substitution for advanced Parkinson’s disease.

**New Approaches**: As with epilepsy, personalized treatment options are being developed for PD, taking into consideration the individual genetic background for disease-modifying therapies [38]. Additionally, there are emerging therapeutic approaches using cannabidiol (CBD), which is also being investigated for its potential use in epilepsy treatment [39].

#### 3.3.2. Deep Brain Stimulation (DBS)

Deep brain stimulation (DBS) has been a well-established therapeutic approach for Parkinson’s disease (PD) since the 1980s [40]. Its positive effects on PD and other movement disorders have been well documented. The primary targets for DBS in PD are the subthalamic nucleus and the globus pallidus internus (GPi), whereas for epilepsy, the target is the anterior nucleus of the thalamus (ANT). DBS has been shown to improve motor symptoms in PD by approximately 40% globally [40]. However, worsening of motor functions has also been reported as a potential side effect. The technique of DBS is continuously improving, particularly with the development of closed-loop DBS, which dynamically adjusts stimulation based on various parameters, especially beta activity [41,42].

## 4. Common Pathophysiology Between PD and Epilepsy

Parkinson’s disease (PD) and epilepsy are both neurological disorders with distinct clinical manifestations. However, emerging research suggests that they may share common pathophysiological mechanisms [10], including the following.

### 4.1. Dopaminergic Dysfunction

PD is characterized by the progressive degeneration of dopaminergic neurons in the substantia nigra pars compacta, leading to both motor and non-motor symptoms. In epilepsy, dopamine has been implicated in seizure modulation. Some studies suggest that dopamine depletion lowers seizure thresholds, making epileptic seizures more likely in PD patients.

### 4.2. Neuroinflammation [43,44,45,46]

Both PD and epilepsy involve chronic neuroinflammation, characterized by the activation of microglia and increased levels of pro-inflammatory cytokines (e.g., IL-1β, TNF-α). In PD, neuroinflammation contributes to dopaminergic neuronal loss, while in epilepsy, it is associated with seizure-induced neuronal damage and epileptogenesis.

### 4.3. Oxidative Stress and Mitochondrial Dysfunction

Mitochondrial dysfunction plays a central role in both disorders. In PD, mitochondrial complex I dysfunction leads to increased reactive oxygen species (ROS) production, causing neuronal apoptosis. In epilepsy, seizures cause excessive ROS production, which contributes to neuronal damage and epileptogenesis.

### 4.4. Excitotoxicity and Glutamate Dysregulation

In PD, excitotoxicity due to glutamate overactivity contributes to dopaminergic neuron degeneration. In epilepsy, excessive glutamatergic activity leads to hyperexcitability and seizure generation.

### 4.5. Genetic Overlaps and Shared Risk Factors

Research suggests that mutations in genes such as LRRK2 and alpha-synuclein (SNCA) are implicated in both PD and epilepsy. Mutations in the LRRK2 gene are well-established risk factors for PD. Notably, the G2019S mutation is prevalent in specific populations, accounting for approximately 20% of PD cases among Ashkenazi Jews and about 40% among North African Berber Arabs. While direct links between LRRK2 mutations and epilepsy are still under investigation, the gene’s role in neuronal signaling and inflammation suggests a possible connection to seizure susceptibility [47].

The SNCA gene encodes α-synuclein, a protein primarily found in the brain that is involved in synaptic function. Mutations and aggregation of α-synuclein are major contributors to PD and related disorders. The accumulation of misfolded α-synuclein protein leads to mitochondrial dysfunction, a common factor in both PD and epilepsy. While SNCA is primarily associated with PD, recent research suggests a link between SNCA dysfunction and epilepsy. α-synuclein regulates neurotransmitter release, thus affecting neuronal excitability, leading to hyperexcitability and increased seizure susceptibility. Reports indicate that patients with SNCA mutations often develop seizures, particularly in the later stages of the disease [48,49,50,51].

The theory that epilepsy and PD might share common genetic mechanisms is intriguing, but there are several limitations in drawing a clear conclusion. The published genetic overlap, whether in variants of PRKN, LRRK2, or GABA, is not extensive enough compared to cases with distinct genetic causes of epilepsy or PD. Both disorders have multiple subtypes with varying genetic contributions and are also influenced by non-genetic factors, such as environmental triggers and epigenetic modifications. These complexities make it challenging to interpret the genetic links and generalize findings across all patients. Furthermore, there is still a lack of large-scale longitudinal studies needed to establish causality.

### 4.6. Altered GABAergic Signaling

GABAergic dysfunction is involved in both diseases. In PD, altered GABAergic signaling in the basal ganglia affects motor control. In epilepsy, GABAergic deficits lead to neuronal hyperexcitability and increased seizure susceptibility.

### 4.7. Aberrant Protein Aggregation

In PD, α-synuclein aggregates (Lewy bodies) disrupt normal neuronal function. Recent studies suggest that α-synuclein may also play a role in epilepsy by altering synaptic function and promoting network hyperexcitability [2].

Although there is potential pathophysiological overlap between these two disorders, whether one could cause the other remains unanswered. Given the complex interactions between genes and cellular pathways—where shared genes often play roles in fundamental cellular processes such as mitochondrial function, autophagy, and neuroinflammation—their contributions to both disorders are not yet fully understood. To address this question, further research is needed to clarify the precise mechanisms linking epilepsy and PD.

## 5. Common Clinical Features Between Epilepsy and PD

### 5.1. Epileptic Seizures and Cortical Hyperexcitability in PD

Both epilepsy and PD exhibit motor dysfunction, though the underlying mechanisms differ [52,53,54]. Among them, 4–6 Hz resting tremors are a hallmark of PD, while postictal tremors and tremor-like myoclonus (e.g., progressive myoclonic epilepsy) can occur in epilepsy [52,53]. The bradykinesia and rigidity typical of PD result from dopaminergic degeneration in the substantia nigra, leading to dopamine loss, which impairs basal ganglia output to the cortex. Similarly, these features can also be observed in epilepsy as postictal bradykinesia and transient rigidity due to temporary disruptions in motor circuits caused by ictal discharges.

While gait disturbances and falls are common in PD, patients with epilepsy may fall due to atonic seizures or postictal instability. Although epilepsy is primarily a disorder of neuronal hyperexcitability, studies suggest that PD patients have a higher seizure risk than the general population [11,43].

The underlying mechanisms may involve dopaminergic neuron degeneration in PD, which could lead to increased cortical excitability [55,56,57]. As mentioned above, these two conditions share many common pathways. Some experimental models have shown that dopamine depletion affects inhibitory neurotransmission, increasing seizure susceptibility. Wise A. et al. (2022) [58] described a case of PD with new-onset refractory myoclonus and focal to bilateral tonic–clonic seizures in a patient who had been treated with Levodopa and Carbidopa for six years. The study revealed that Vitamin B6 deficiency under high levodopa dosages might have caused this seizure condition. Further animal studies suggest that levodopa may influence seizure susceptibility via GABAergic interactions with the nigrostriatal dopaminergic system [59].

### 5.2. Cognitive Dysfunction in Epilepsy and PD

Both PD and epilepsy are neurological disorders that can lead to cognitive dysfunction. While they have different pathophysiological mechanisms, they share some common cognitive impairments. Both disorders involve hippocampal and frontal lobe dysfunction, leading to memory loss and executive dysfunction. In temporal lobe epilepsy (TLE), repeated seizures cause hippocampal atrophy, resulting in memory impairments. PD-related cognitive impairment progresses from mild cognitive impairment (PD-MCI) to Parkinson’s disease dementia (PDD), similar to epileptic dementia syndromes [60].

#### 5.2.1. Memory Impairment

Patients with PD and epilepsy often experience deficits in working memory and episodic memory. In PD, memory dysfunction is linked to dopamine depletion in the striatum and frontal lobe dysfunction [60]. In epilepsy, particularly TLE, memory issues arise from hippocampal sclerosis and network disruption [61].

#### 5.2.2. Executive Dysfunction

Impairments in planning, problem-solving, and cognitive flexibility are common in both conditions. Studies have shown that PD-related executive dysfunction is due to basal ganglia-thalamo-cortical circuit disruption [62]. Epilepsy-related executive dysfunction is observed in patients with frontal lobe epilepsy and is due to seizure-related neuronal loss [63,64].

#### 5.2.3. Attention Deficits

Sustained and selective attention deficits are reported in both disorders. In PD, attentional deficits are associated with cholinergic system dysfunction [65]. In epilepsy, attention deficits can result from interictal discharges affecting cortical networks [66].

### 5.3. Neuropsychiatric Symptoms

Both conditions exhibit high rates of psychiatric comorbidities due to dopaminergic, serotonergic, and GABAergic dysfunction.

#### 5.3.1. Depression and Anxiety

Depression and anxiety are very common in patients with PD, likely due to dopaminergic and serotonergic dysfunction [67]. A higher prevalence is observed in epilepsy, likely due to limbic system involvement [68].

#### 5.3.2. Hallucinations and Psychosis

In PD, hallucinations and psychosis may be medication-induced (dopaminergic therapy) or disease-related (Lewy body pathology). In epilepsy, postictal psychosis often follows complex partial seizures [69,70].

#### 5.3.3. Sleep Disturbances

Sleep disturbances are also very common in both PD and epilepsy. In PD, they often manifest as REM sleep behavior disorder (RBD), insomnia, and excessive daytime sleepiness [71]. In epilepsy, nocturnal seizures can disrupt sleep architecture [72].

### 5.4. Autonomic Dysfunction

Both disorders can cause dysautonomia, such as orthostatic hypotension, due to autonomic failure in PD and occurring postictally or as a side effect of medications in epilepsy [73,74]. Additionally, gastrointestinal dysfunction, such as constipation, is very common in PD [75] and may also be associated with autonomic seizures [76].

While epilepsy and Parkinson’s disease have distinct primary pathologies—seizure generation in epilepsy and dopaminergic neurodegeneration in PD—there is significant overlap in motor, cognitive, neuropsychiatric, and autonomic symptoms. The shared features suggest common underlying neurobiological mechanisms, such as neuroinflammation and mitochondrial dysfunction.

## 6. Common Therapy Between PD and Epilepsy

Parkinson’s disease (PD) and epilepsy are both neurological disorders with distinct pathophysiologies. However, they share overlapping mechanisms, such as neuroinflammation, oxidative stress, and neurotransmitter imbalances, which have led to the exploration of common therapeutic strategies. Below are some of the shared treatment approaches.

### 6.1. Pharmacological Therapies

#### 6.1.1. Dopaminergic Agents

The impact of dopamine agonists on seizure control has been studied for over a century. Many studies suggest the anticonvulsive effects of dopamine agonists. Turski WA (1990) [77], Starr (1996) [78] and Brodovskaya (2021) [79] published findings on the anticonvulsive effects of multiple dopamine receptor families (D1 and D2), showing that they mediate opposing influences on neuronal excitability. These findings may open a new era in dopamine–epilepsy research.

#### 6.1.2. Antiseizure Medication (ASMs)

Some ASMs may be beneficial for both conditions. For example, lamotrigine (LTG) has mood-stabilizing properties and may provide neuroprotection in PD. Levetiracetam (LEV), due to its minimal drug interactions, is useful in PD patients with epilepsy. Conversely, the use of valproic acid (VPA) should be carefully evaluated in PD patients due to its risk of worsening parkinsonian symptoms.

### 6.2. Neuroprotective Strategies

Antioxidants (e.g., Coenzyme Q10, Vitamin E) may help reduce oxidative stress, which is implicated in both PD and epilepsy. Additionally, anti-inflammatory drugs (e.g., NSAIDs, Minocycline) may help modulate neuroinflammation, a factor contributing to disease progression in both conditions.

### 6.3. Deep Brain Stimulation (DBS)

As previously mentioned, subthalamic nucleus (STN) stimulation has been proven effective for motor symptoms in PD and has also shown promise in reducing seizure frequency in epilepsy patients. Additionally, anterior thalamic nucleus (ATN) stimulation, which is used for drug-resistant epilepsy, may provide cognitive benefits in PD [41,80].

### 6.4. Lifestyle and Alternative Therapies

Several other therapeutic methods are used for both conditions. For example, the **Ketogenic Diet**, traditionally used in epilepsy, is being explored for PD due to its potential neuroprotective effects [81]. Additionally, **Exercise and Physiotherapy** are beneficial in both conditions for maintaining motor function and promoting neuroplasticity [82,83].

## 7. Discussion

Epilepsy and movement disorders are two major categories of neurological conditions that can coexist, overlap, or mimic each other, posing diagnostic and therapeutic challenges. This review provides a comprehensive overview of both conditions, their intersection, and the current literature on their pathophysiology, diagnosis, and management.

In a large retrospective cohort study, the association between incident Parkinson’s disease (PD) and subsequent incident epileptic seizures was investigated using a nested case–control analysis. Data from 23,086 patients aged ≥40 years from the U.K. Clinical Practice Research Datalink with incident Parkinson’s disease between 1996 and 2016, along with a matched comparison group of 92,343 PD-free individuals, were analyzed [11]. PD was defined as the first recorded medical diagnosis (Read-code) according to the International Classification of Diseases Version 10 (ICD-10) code G20 (Parkinson’s disease), while epileptic seizures or epilepsy were identified using ICD-10 codes G40 (“Epilepsy”), G41 (“Status epilepticus”), or R56.8 (“Other and unspecified convulsions”). Crude incidence rates (IRs) with 95% confidence intervals (CIs) of epileptic seizures in PD patients and the PD-free comparison group were calculated, along with crude incidence rate ratios (IRRs) and adjusted odds ratios (adj. ORs). The adjusted ORs for incident PD among cases with incident epileptic seizures and seizure-free controls were further stratified by various seizure-provoking comorbidities, such as brain disorders, psychiatric disorders, metabolic disorders, dementia, and substance abuse. A total of 284 (1.2%) PD patients had incident epileptic seizures, compared to 614 (0.7%) patients in the PD-free control group. The crude overall IRR of epileptic seizures in PD patients compared to the PD-free control group was 2.37 (95% CI: 2.06–2.37), with higher incidence rates in older individuals in both groups. After adjusting for potential confounders, a 1.7-fold increased risk of epileptic seizures in PD patients compared to PD-free individuals was observed (adjusted OR: 1.68, 95% CI: 1.43–1.98). PD patients without seizure-provoking comorbidities had a 2.2-fold higher risk of epileptic seizures. Patients with comorbid brain disorders (vascular risk factors, dementia, or multiple seizure-provoking comorbidities) had a significantly higher risk of epileptic seizures (10–13 times higher) compared to PD-free individuals without any seizure-provoking comorbidities. These findings suggest that PD alone is associated with an increased risk of incident epileptic seizures, with PD patients who have proconvulsive comorbidities being at even greater risk than PD-free individuals with similar comorbidities. Although selection bias was present, this study represents the largest longitudinal clinical observation of the incidence of epileptic seizures in PD with stratification by seizure-provoking comorbidities. The results were considered convincing. Similar result was also suggested in a recent study [84]. 

It has been proposed that common risk factors for structural brain damage may underlie the increased coincidence of these two diseases. Another potential factor linking epilepsy and PD is inflammation [43]. Karceski et al. described inflammatory pathophysiological mechanisms as an important risk factor for the interplay between epilepsy and Parkinson’s disease. Several publications in recent decades support the hypothesis that inflammatory mechanisms contribute significantly to epilepsy [16,43]. For PD, inflammatory mechanisms also play a crucial role. Calcium (Ca^2+^) dysregulation is a key pathophysiological concept explaining inflammatory processes, where cytokine activation mediates inflammation in the context of both diseases. The use of amlodipine, a calcium channel blocker, has demonstrated anti-inflammatory effects by reducing intracellular calcium levels. During the COVID-19 pandemic, this therapeutic approach showed promising results in significantly reducing elevated calcium levels caused by COVID-19 infections [47,85].

Another experimental therapeutic approach to reducing inflammation-based seizures has involved using interleukin-1β antagonists [86], which have demonstrated seizure-reducing effects in inflammation-based epilepsies. However, the relationship between inflammatory processes and PD remains less clear than in epilepsy. Epidemiological data indicating a higher risk of PD following intracranial trauma suggest that inflammatory pathomechanisms may also play a role in PD development. It is believed that calcium dyshomeostasis can lead to dopaminergic neuron degeneration, triggering uncontrolled inflammatory reactions and potentially contributing to PD occurrence [43].

Deransart et al. [87] suggested that dysfunction of dopaminergic transmission is not considered a primary cause of epilepsy. However, one hypothesis is that basal ganglia modulation of seizure activity via thalamic networks could lead to uncontrolled seizure activity.

On the other hand, some studies report conflicting findings regarding the association between epilepsy and PD. Some publications and case reports even indicate a lower prevalence of seizures in PD patients [88,89,90,91]. Feddersen et al. [91] compared 1215 patients with idiopathic PD, 31 with concomitant epilepsy, and 2537 epilepsy patients without PD (mean age: 43 years). They analyzed the incidence of epilepsy and status epilepticus in PD patients compared to those without PD. Their findings showed a significantly increased rate of status epilepticus in idiopathic PD patients compared to epilepsy patients. However, consistent with previous studies, the overall prevalence of epileptic seizures in PD patients was lower than expected for their age group. In their study, 2.6% of PD patients had epilepsy, similar to the previously reported 2.4% [89,91]. When calculating the prevalence of each disorder, this equated to only one or two cases per 100,000 individuals over the age of 60. Despite the significantly higher risk of status epilepticus in elderly PD patients, these studies primarily relied on cross-sectional data without matched comparison groups and lacked adjustment for confounding factors, limiting their interpretative power.

In summary, the true relationship between epilepsy and PD remains unclear. Most findings are based on retrospective observational studies with varying diagnostic criteria, selection biases, and, in some cases, small sample sizes. Therefore, any interpretation of these results should be approached with caution.

## 8. Conclusions

Epilepsy and Parkinson’s disease (PD) are both neurological disorders with distinct causes and mechanisms. However, research suggests there may be links between the two. They may share common pathophysiological features and some genetic abnormalities involving abnormal brain activity. Neuroinflammation, oxidative stress, and mitochondrial dysfunction have been implicated in both disorders. Studies suggest that people with PD may have a higher risk of developing epilepsy, particularly in the later stages of the disease, possibly due to neurodegeneration, reduced brain plasticity, and structural changes in the brain. Parkinsonism has also been observed in patients with epilepsy. Some anti-seizure drugs can worsen Parkinsonian symptoms by affecting dopamine metabolism. Conversely, certain anti-Parkinson’s medications, such as dopamine agonists, might lower the seizure threshold and increase the risk of seizures.

While epilepsy and Parkinson’s disease are separate conditions, they share overlapping mechanisms, and one may increase the risk of the other. Although some observational studies exist, more convincing data from meta-analyses and randomized controlled trials are still lacking. To clarify the relationship, the underlying mechanisms, and to provide therapeutic guidelines, better-designed studies are needed. Nevertheless, greater attention should be paid to patients with PD to avoid both the underdiagnosis of epileptic seizures and the inappropriate application of anti-seizure medications (ASMs).
